# Contrasting anatomical and biochemical controls on mesophyll conductance across plant functional types

**DOI:** 10.1111/nph.18363

**Published:** 2022-08-02

**Authors:** Jürgen Knauer, Matthias Cuntz, John R. Evans, Ülo Niinemets, Tiina Tosens, Linda‐Liisa Veromann‐Jürgenson, Christiane Werner, Sönke Zaehle

**Affiliations:** ^1^ Hawkesbury Institute for the Environment Western Sydney University Penrith NSW 2751 Australia; ^2^ Climate Science Centre CSIRO Oceans and Atmosphere Canberra ACT 2601 Australia; ^3^ Max Planck Institute for Biogeochemistry 07745 Jena Germany; ^4^ AgroParisTech, UMR Silva INRAE, Université de Lorraine 54000 Nancy France; ^5^ ARC Centre of Excellence for Translational Photosynthesis Research School of Biology The Australian National University Canberra ACT 2601 Australia; ^6^ Institute of Agricultural and Environmental Sciences Estonian University of Life Sciences 51006 Tartu Estonia; ^7^ Ecosystem Physiology, University of Freiburg 79110 Freiburg Germany

**Keywords:** leaf anatomy, leaf internal CO_2_ transfer, leaf gas exchange, leaf nutrient content, photosynthetic capacity, photosynthetic limitation

## Abstract

- Mesophyll conductance (*g*_m_) limits photosynthesis by restricting CO_2_ diffusion between the substomatal cavities and chloroplasts. Although it is known that *g*_m_ is determined by both leaf anatomical and biochemical traits, their relative contribution across plant functional types (PFTs) is still unclear.
- We compiled a dataset of *g*_m_ measurements and concomitant leaf traits in unstressed plants comprising 563 studies and 617 species from all major PFTs. We investigated to what extent *g*_m_ limits photosynthesis across PFTs, how *g*_m_ relates to structural, anatomical, biochemical, and physiological leaf properties, and whether these relationships differ among PFTs.
- We found that *g*_m_ imposes a significant limitation to photosynthesis in all C_3_ PFTs, ranging from 10–30% in most herbaceous annuals to 25–50% in woody evergreens. Anatomical leaf traits explained a significant proportion of the variation in *g*_m_ (*R* ^2^ > 0.3) in all PFTs except annual herbs, in which *g*_m_ is more strongly related to biochemical factors associated with leaf nitrogen and potassium content.
- Our results underline the need to elucidate mechanisms underlying the global variability of *g*_m_. We emphasise the underestimated potential of *g*_m_ for improving photosynthesis in crops and identify modifications in leaf biochemistry as the most promising pathway for increasing *g*_m_ in these species.

## Introduction

The supply of CO_2_ to the photosynthetic machinery depends on how efficiently it can be transferred from the ambient air to the chloroplasts located inside the leaf mesophyll cells. This efficiency can be quantified as a series of resistances (or the inverse quantity, conductances) caused by the leaf boundary layer, the stomata, as well as leaf internal components in the mesophyll. This last part of the CO_2_ pathway, the mesophyll conductance (*g*_m_), accounts for one‐third to one‐half of the overall CO_2_ drawdown from the atmosphere to the chloroplasts (Warren, 2008; Flexas *et al*., 2012) and therefore constitutes a major controlling factor of the CO_2_ concentration available for photosynthesis. Knowledge of the determinants of *g*_m_ can therefore support efforts aiming to improve photosynthesis to ensure that global food and bioenergy demand can be met in the future (von Caemmerer & Evans, 2010; Ort *et al*., 2015). Furthermore, information of how *g*_m_ is related to key leaf structural and biochemical traits is important for understanding and modelling carbon uptake from the leaf to the global scale (Niinemets *et al*., 2009; Sun *et al*., 2014; Knauer *et al*., 2019, 2020).

The pathway of CO_2_ within plant leaves can be divided into several components, which in combination determine the magnitude of *g*_m_: the intercellular airspaces, the cell wall, the plasma membrane, the cytosol, the chloroplast envelope, and the chloroplast stroma (Niinemets & Reichstein, 2003; Evans *et al*., 2009). Some of the conductances within these components depend primarily on biophysical characteristics (e.g. surface area of chloroplasts exposed to intercellular airspaces, cell wall thickness and porosity) and are therefore subject to anatomical constraints, whereas CO_2_ transfer through other cell compartments such as membranes and the cytosol are primarily the result of biochemical factors, in particular the expression of proteins associated with CO_2_ transfer. These include aquaporins (cooporins), proteins that regulate water and CO_2_ transfer across membranes (Uehlein *et al*., 2003), and carbonic anhydrase (CA), which governs the interconversion between CO_2_ and bicarbonate in the cytosol and chloroplast stroma (Fabre *et al*., 2007; Evans *et al*., 2009). Despite the fact that it is well established that *g*_m_ is affected by both anatomical and biochemical leaf traits (Warren, 2008; Flexas *et al*., 2012, 2018; Gago *et al*., 2020), their relative contribution across plant functional types (PFTs) has not yet been assessed.

The complexity of the CO_2_ diffusion pathway within leaves results in considerable uncertainties regarding the contributions of the individual components to the overall conductance as well as the associated importance of key anatomical and biochemical traits. One possible avenue to elucidate the role of certain leaf traits in determining *g*_m_ are gas diffusion models that calculate the component conductances based on biophysical and biochemical principles (Niinemets & Reichstein, 2003; Tomás *et al*., 2013; Berghuijs *et al*., 2015; Xiao & Zhu, 2017). However, these models either do not take all relevant mechanisms into account (e.g. biochemistry, location of individual elements of the diffusion pathway) or require parameters that are unknown or only available for a few species, which hinders the interpretation of these models as well as their application across PFTs.

An alternative approach followed by many studies is to use correlation analysis to investigate to what extent *g*_m_ measurements are related to leaf anatomical and biochemical traits. However, most studies are restricted to one or a few species of the same PFT and are subject to differences in growth environments, measurement conditions, as well as assumptions and uncertainties inherent in different measurement approaches (Pons *et al*., 2009). These differences can hamper a direct comparison between individual studies and preclude robust conclusions. In addition, correlations can only provide associative rather than causal relationships between *g*_m_ and leaf traits. Despite these limitations, a correlative approach can provide information about key traits covarying with *g*_m_ and therefore highlight the trait syndromes responsible for the variation in *g*_m_, especially if relationships emerge across studies, species, and conditions (e.g. Xiong & Flexas, 2018; Ren *et al*., 2019; Elferjani *et al*., 2021).

Here, we present the hitherto largest published dataset of *g*_m_ measurements compiled from the literature (comprising 563 studies). We performed a comprehensive analysis that aimed to investigate the relationships between *g*_m_ and accompanying leaf structural, anatomical, biochemical and physiological traits measured on the same set of plants. The overarching goal of this top‐down approach was to identify patterns between *g*_m_ and leaf traits that are robust with respect to existing confounding effects of different species and genotypes, growth conditions, or methodological considerations, and that may guide future research priorities. In particular, we asked (1) how much *g*_m_ limits photosynthesis across PFTs, (2) to what extent leaf anatomical and biochemical factors can explain variations in *g*_m_ across and within PFTs, and (3) how our findings could be used to enhance *g*_m_ and photosynthesis.

## Materials and Methods

### Literature review

A literature review was conducted in Google Scholar using the search terms ‘mesophyll conductance’ and ‘leaf internal conductance’. All peer‐reviewed studies that were published online until 31 December 2020 were considered. Criteria for inclusion into the dataset were that *g*_m_ was estimated at leaf level using any published method and that it was defined according to Fick's first law as *g*_m_ = *A*_n_/(*C*_i_ − *C*_c_), where *A*_n_ is net photosynthesis, *C*_i_ is the intercellular CO_2_ concentration, and *C*_c_ is the chloroplastic CO_2_ concentration. No modelled *g*_m_ data were included. *g*_m_ values and all accompanying traits presented here were extracted from tables or the text, if possible, otherwise digitised from figures using plotdigitizer v.2.6.8 (http://plotdigitizer.sourceforge.net/). The compilation aimed to represent unstressed, young, but fully expanded and high light‐adapted leaves, albeit these criteria were not always explicitly stated. In studies including treatments, only data from the control treatment were extracted. Only one (aggregated) *g*_m_ value per set of plants was included in the dataset.

### Data processing

#### Mesophyll conductance

Mesophyll conductance values were standardised to represent *g*_m_ to CO_2_ transfer in units of mol m^−2^ s^−1^. Values reported in liquid‐phase equivalent units (mol m^−2^ s^−1^ bar^−1^ or μmol m^−2^ s^−1^ Pa^−1^) were standardised to an atmospheric pressure of 100 kPa (=1 bar) if either the atmospheric pressure or the elevation (from which mean atmospheric pressure was derived) were reported, otherwise an atmospheric pressure of 100 kPa was assumed. Measurements not performed at 25°C or not standardised to 25°C in the original studies were standardised to 25°C (denoted as *g*_m,25_) using the temperature response of Bernacchi *et al*. (2002) measured for *Nicotiana tabacum*. The functional shape of this temperature function was confirmed by an independent study over a wide temperature range (Evans & von Caemmerer, 2013). To characterise the degree of uncertainty associated with the temperature response of *g*_m_, the analysis was also performed using a weaker temperature response derived for *Arabidopsis thaliana* (Walker *et al*., 2013). Values at the original measurement temperature (denoted as *g*_m_) were retained in the dataset and reported here if shown together with other physiological measurements conducted at the same temperature.

Measurements were discarded if they met one or more of the following criteria: (1) measurement temperature lower than 15°C or higher than 35°C, or not reported; (2) measurement irradiance lower than 300 μmol m^−2^ s^−1^; (3) measurement CO_2_ concentration lower than 300 μmol mol^−1^ or higher than 500 μmol mol^−1^; (4) measurements associated with unrealistic CO_2_ drawdown values according to Fick's first law (*C*_i_ − *C*_c_ = *A*_n_/*g*_m_ greater than 300 μmol mol^−1^ or smaller than 10 μmol mol^−1^); and (5) values identified as outliers. Outliers were detected with a two‐step procedure: first, extreme values exceeding 2 mol m^−2^ s^−1^ or 1 mol m^−2^ s^−1^ for herbaceous and woody plants, respectively, were excluded. Second, the remaining data were log‐transformed and all data lower than the first quartile minus 1.5 times the interquartile range (IQR) and higher than the third quartile plus 1.5 times the IQR were excluded. Step two was performed separately for each PFT. Sixty‐one outliers were detected across the dataset. In total, data filtering led to the exclusion of 200 datapoints that left 1683 data points (89.4%) from 492 studies (87.4%) for subsequent analysis.

All published methods for estimating *g*_m_ were considered for the analysis (Supporting Information Fig. S1). If *g*_m_ was measured with both the curve fitting and a second method, only the second method was used for the analysis. If *g*_m_ was measured with two methods other than curve fitting, *g*_m_ was calculated as the mean of the two methods. The associated averaging of *g*_m_ measurements across methods decreased the available data by another 244 data points.

Species were grouped into the following major PFTs according to their evolutionary lineage and growth habits (leaf longevity): ferns, evergreen gymnosperms, woody evergreen angiosperms, woody deciduous angiosperms, C_3_ perennial herbaceous, C_3_ annual and biennial herbaceous (from this point forwards C_3_ annual herbaceous), in which herbaceous includes both forbs and grasses. The dataset also contains values for Crassulacean acid metabolism (CAM) plants, C_4_ plants (both annual and perennial herbaceous), semideciduous angiosperms, deciduous gymnosperms, as well as fern allies and mosses, but these PFTs (in total 102 data points (7.1%) after data filtering) were not included in this analysis due to limited data availability. Excluding these PFTs from the dataset left 1337 out of 1883 datapoints (71.0%) from 476 studies (84.5%) and 495 species (80.2%) available for analysis in this study.

#### Accompanying traits and variables

In addition to *g*_m_, leaf physiological, structural, anatomical and biochemical traits and variables, as well as ancillary information such as measurement method, measurement and growth conditions, plant age, etc. were extracted from the studies (please refer to Table S1 for a full list and the full dataset (Knauer *et al*., 2022) for additional traits and variables not presented here). All observations for a given trait were converted to a common unit as specified in Table S1. Care was taken that all extracted values were measured in the same experiments and treatments as the presented *g*_m_ values. That means that all traits analysed here were measured in the same set of plants subject to the same experimental treatment, growth conditions and measurement conditions.

#### Photosynthetic limitation

Relative photosynthetic limitation caused by *g*_m_ (*L*_m_) was originally proposed by Farquhar & Sharkey (1982) for stomatal conductance (*g*_s_) and subsequently applied to *g*_m_ (Epron *et al*., 1995; Warren *et al*., 2003):

(Eqn 1) $$L_{m}=\frac{A_{\mathrm{np}}-A_{n}}{A_{\mathrm{np}}}\times 100\;$$

where *A*_n_ is the light‐saturated net photosynthesis measured at ambient CO_2_ concentration (i.e. assuming *g*_m_ and *g*_s_ as measured), and *A*_np_ is the net photosynthesis at *C*_c_ = *C*_i_ (i.e. assuming infinite *g*_m_ and *g*_s_ as measured). As most studies did not report all parameters needed to calculate *L*_m_, data analysed here were limited to those directly reported in the studies. *L*_m_ as defined in Eqn 1 was preferred over the limitation analysis suggested by Grassi & Magnani (2005) because it allows inferences on the absolute limitation of *A*_n_ by *g*_m_, whereas the method by Grassi & Magnani (2005) quantifies the photosynthetic limitation of *g*_m_ relative to those imposed by *g*_s_ and photosynthetic capacity.

### Statistical analysis

Pairwise relationships between *g*_m_ and leaf traits were characterised with robust linear or robust nonlinear regressions using the robustbase R package (Maechler *et al*., 2022). Differences in the median among groups was tested with Dunn's test of multiple comparisons, using the *dunnTest* function in the R package fsa (Ogle *et al*., 2022). Statistical significance (*P* < 0.05) of the relationships was only tested and reported if the number of measurements were ≥ 12, unless stated otherwise.

As *g*_m_ data show a gamma distribution rather than a normal distribution, linear regression models are not ideal for modelling *g*_m_. Therefore, to predict *g*_m_ from anatomical traits we applied a generalised linear model (glm) with a gamma error distribution and a log‐link function. To assess glm model fits, McFadden's pseudo *R* ^2^ was calculated as *R* ^2^ = 1 − $\frac{\ln L\left(M_{\text{Full}}\right)}{\ln L\left(M_{\text{Null}}\right)}$, where ln *L*(*M*_full_) is the log‐likelihood of the full model (i.e. all coefficients fitted) and ln *L*(*M*_Null_) is the log‐likelihood of the null model (i.e. only intercept fitted). All data processing and statistical analysis was conducted in R v.4.1.2 (R Core Team, 2021).

## Results

**Fig. 1:**
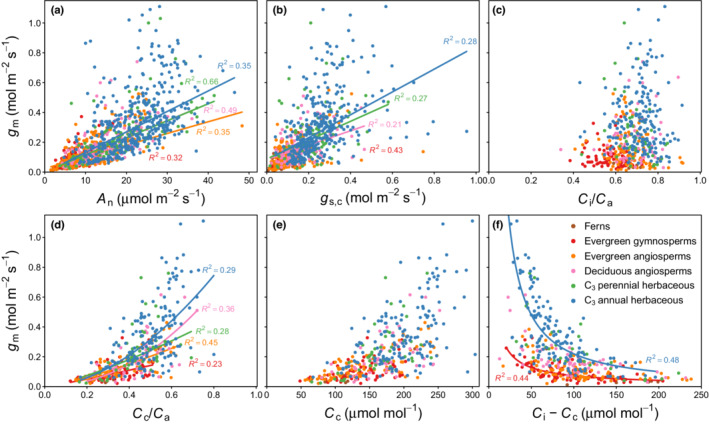
Relationship between mesophyll conductance (*g*_m_) and leaf gas exchange variables: (a) net photosynthesis (*A*_n_), (b) stomatal conductance to CO_2_ (*g*_s,c_), (c) the ratio of intercellular CO_2_ concentration (*C*_i_) to ambient CO_2_ concentration (*C*_a_), (d) the ratio of chloroplastic CO_2_ concentration (*C*_c_) to *C*_a_, (e) *C*_c_, and (f) the CO_2_ drawdown between intercellular airspaces and the chloroplast stroma (*C*_i_ − *C*_c_). Robust linear regression was applied in panels (a, b) and robust nonlinear regression of the form *y* = *ax* ^*b*^ in all other panels. Model fits and corresponding *R* ^2^ values are displayed if the model *P*‐value < 0.05.

After data filtering 1337 individual *g*_m_ values from 476 studies representing all published methods on estimating *g*_m_ were left for analysis (please refer to Fig. S1 for the number of studies per year and method used for estimating *g*_m_). The data show typical relationships between *g*_m_ and other leaf gas exchange variables (Fig. 1). We found moderate correlations between *g*_m_ and light‐saturated net photosynthesis (*A*_n_) and to a lesser extent also between *g*_m_ and stomatal conductance to CO_2_ (*g*_s,c_). Generally, a stronger relationship was observed for herbaceous plants and deciduous angiosperms compared with woody species for both *A*_n_ and *g*_s,c_ (Fig. 1a,b). *g*_m_ does not show a clear relationship with the ratio of intercellular CO_2_ concentration (*C*_i_) to ambient CO_2_ concentration (*C*_a_) but a positive relationship with the chloroplastic CO_2_ concentration (*C*_c_) and the *C*_c_ : *C*_a_ ratio. There is furthermore a clear inverse relationship between *g*_m_ and the CO_2_ drawdown (*C*_i_ − *C*_c_) across PFTs, in which herbaceous annuals tend to show the highest CO_2_ drawdown for a given *g*_m_ (Fig. 1f).

**Fig. 2:**
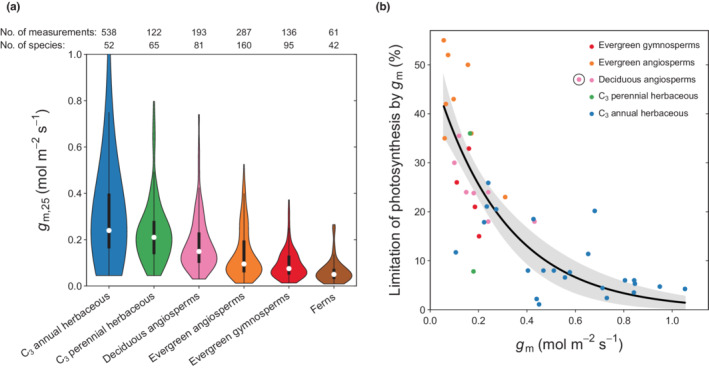
(a) Violin plots showing the distribution of mesophyll conductance measurements standardised to 25°C (*g*_m,25_) for main plant functional types (PFTs). Dots represent group medians, black bars represent the interquartile range, and black lines represent 1.5 times the interquartile range. All PFT medians were statistically significant from each other according to Dunn's test. (b) Limitation of leaf photosynthesis by *g*_m_ across plant functional types. Photosynthetic limitation (*L*_m_) was calculated as (*A*_np_ *− A*_n_)/*A*_np_) × 100 (Eqn 1), where *A*_n_ is measured net photosynthesis and *A*_np_ is net photosynthesis assuming *C*_i_ = *C*_c_ (please refer to the Materials and Methods section). The fitted line represents the nonlinear regression *L*_m_ = 50.2 exp(−3.37 *g*_m_) (*R* ^2^ = 0.70, *P* < 0.001). The circled point (*Prunus persica*) was excluded from the regression. The shaded area represents the 95% confidence interval of the regression fit.

Mesophyll conductance standardised to 25°C (*g*_m,25_, please refer to the Materials and Methods section) is higher in herbaceous species than in woody species and higher in species with annual leaves compared with those with long‐lived leaves in both herbaceous and woody plants. Ferns showed the lowest *g*_m_ values of all PFTs (Fig. 2a). Absolute values of *g*_m,25_ depend on the standardisation function used, which also affects statistical relationships found here (please refer to Table S2). However, differences were mostly minor or limited to individual PFTs and therefore did not affect key results. We also tested whether different measurement methods (fluorescence, isotope, curve fitting and others (please refer to e.g. Pons *et al*., 2009 for an overview of the different methods)) predict different magnitudes of *g*_m_. While we find differences among methods for some PFTs and a general tendency of the isotope method to yield higher values compared with the fluorescence and curve fitting method, statistically significant differences among methods could only be detected for some PFTs (Fig. S2).

We investigated to what extent the measured values of *g*_m_ limit photosynthesis (Fig. 2b). We compiled data representing the relative limitation of *A*_n_ by *g*_m_ (*L*_m_; Eqn 1) (Farquhar & Sharkey, 1982; Epron *et al*., 1995), a metric quantifying to what extent *A*_n_ could be enhanced if *g*_m_ was infinitely high (please refer to the Materials and Methods section). We found that at ambient CO_2_ concentrations *g*_m_ imposes a significant limitation to photosynthesis in most plants (Fig. 2b). *L*_m_ increases sharply with decreasing *g*_m_ and reaches 35–55% if *g*_m_ is smaller than 0.1 mol m^−2^ s^−1^. At the higher end of the *g*_m_ range *L*_m_ approaches 0. Using the 95% confidence interval of the fitted function in Fig. 2(b) to predict *L*_m_ from a typical range (25^th^ to 75^th^ quantile) of measured *g*_m_ values as shown in Fig. 2(a) suggests that the limitation of photosynthesis by *g*_m_ amounts to 29–49% in evergreen gymnosperms, 23–47% in evergreen angiosperms, and 20–40% in deciduous angiosperms for representative plants in each PFT (i.e. those with *g*_m_ in the interquartile range; Fig. 2a). Individual *L*_m_ values may be well above or below the PFT‐specific averages (Fig. 2b) and the ranges of *L*_m_ across PFTs overlap to a large extent, reflecting the large spread of *g*_m_ values within PFTs (Fig. 2a). In addition, as *L*_m_ depends not only on the absolute value of *g*_m_ but also on *g*_s_ and leaf photosynthetic capacity (foremost the maximum carboxylation rate (*V*_cmax_)), interspecific variations in these two variables are likely to contribute to the scatter in Fig. 2(b) (please refer to e.g. fig. 1 in Epron *et al*., 1995). *L*_m_ is lower in herbaceous plants but still amounted to 16–35% and 9–32% in representative perennial and annual herbs, respectively. Notably, photosynthetic limitations of < 10% are typically only present if *g*_m_ exceeds *c*. 0.5 mol m^−2^ s^−1^, a value that is commonly not reached even in annual herbaceous plants, which include most crops (Figs 2a, S3). The fitted function in Fig. 2(b) suggests a limitation of 20.9% (95% confidence interval = (17.6, 24.2)%) for an average crop species (median *g*_m,25_ = 0.26 mol m^−2^ s^−1^, Fig. S3) and higher values in species with low *g*_m,25_ such as rice (*Oryza sativa*; 0.23 mol m^−2^ s^−1^) and bean (*Phaseolus vulgaris*; 0.24 mol m^−2^ s^−1^).

We next analysed which leaf traits determine absolute values of *g*_m_. We did not find significant relationships between the magnitude of *g*_m_ and commonly measured leaf structural traits. Leaf dry mass per area (LMA), leaf thickness, mesophyll thickness, leaf density, and leaf porosity were not related to *g*_m_ neither across nor within PFTs (Fig. S4). Stomatal characteristics (stomatal density, area, and length) were in general unrelated to *g*_m_ but *g*_m_ in annual herbaceous plants showed a significant (*P* < 0.05) decrease with stomatal length and area, and a significant increase with stomatal density, although correlations were generally weak (Fig. S4).

**Fig. 3:**
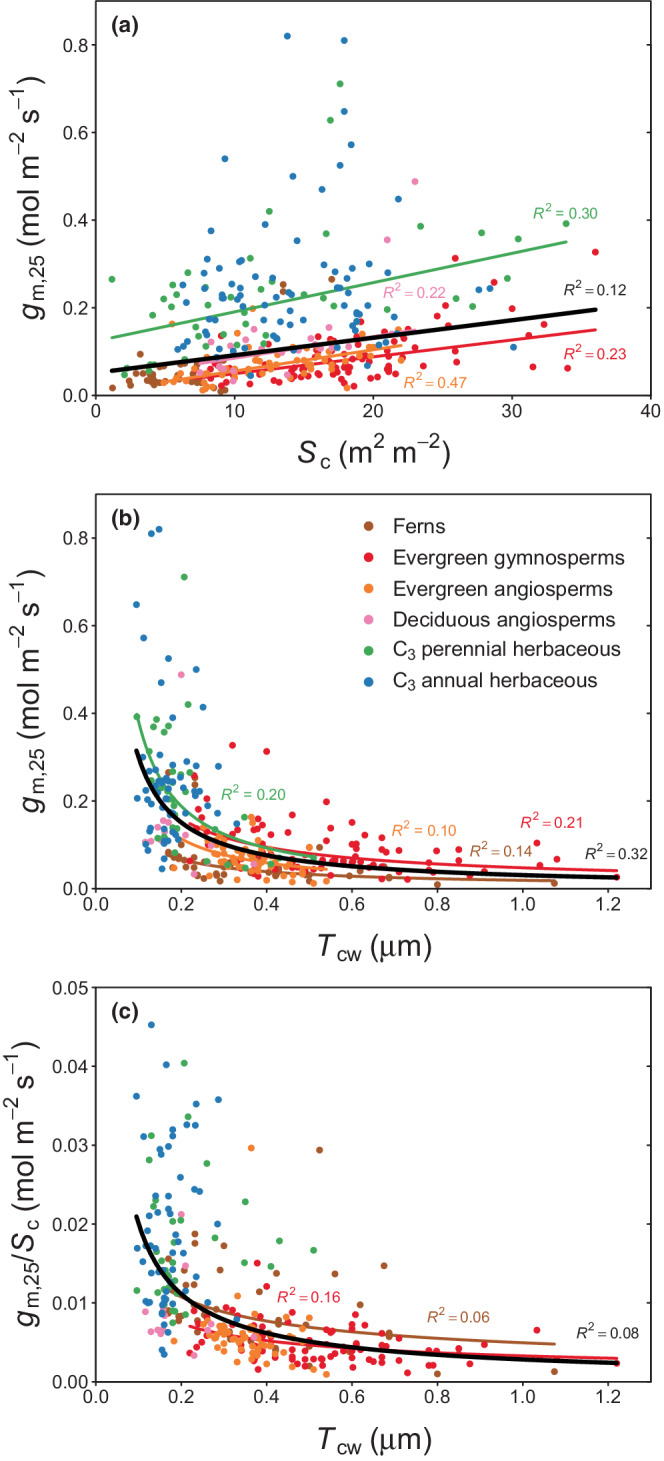
Mesophyll conductance standardised to 25°C (*g*_m,25_) across plant functional types (PFTs) in relation to (a) chloroplast surface area exposed to the intercellular airspaces per unit leaf area (*S*_c_), (b) cell wall thickness (*T*_cw_), and (c) *g*_m,25_ normalised by *S*_c_ in relation to *T*_cw_. Lines represent robust linear regression fits (panel a) and nonlinear regression fits of the form *y* = *ax* ^*b*^ (panels b, c), respectively, and were drawn if *P* < 0.05. Black lines represent global regression fits (all PFTs pooled).

Two anatomical traits were found to play a significant role for *g*_m_ across PFTs: chloroplast surface area exposed to the intercellular airspaces per unit leaf area (*S*_c_), a measure for the surface area available for direct CO_2_ exchange between the intercellular airspaces and the chloroplasts, and cell wall thickness (*T*_cw_) (Fig. 3a,b). The mesophyll cell surface area facing the intercellular airspaces (*S*_m_) also relates to *g*_m_, but the relationship was generally weaker with *S*_m_ than with *S*_c_ (Fig. S5a). Across PFTs, larger values of *g*_m_ are typically associated with a large *S*_c_ and thinner cell walls (a small *T*_cw_) and show a linear increase with *S*_c_ and a nonlinear decrease with *T*_cw_ (Fig. 3a,b). For a given *S*_c_, herbaceous plants show a higher *g*_m_ than other PFTs (Fig. 3a; Table S2). When looking at the mesophyll conductance per unit exposed chloroplast surface area (*g*_m,25_/*S*_c_) (Fig. 3c), it becomes apparent that the conductance per unit exposed chloroplast surface area decreases with increasing *T*_cw_. Herbaceous PFTs have a larger *g*_m_ per unit *S*_c_ compared with woody plants or ferns. Although a significant relationship exists across PFTs, strong relationships within PFTs are generally missing, indicating that *T*_cw_ does not explain a large amount of the variation in *g*_m_ if differences in *S*_c_ are accounted for. Most other leaf anatomical traits commonly reported in studies were not related to *g*_m_: cytosol thickness (*T*_cytosol_) and chloroplast thickness (*T*_chloroplast_), as well as metrics describing chloroplast dimensions that did not show a statistically significant relationship with *g*_m_ (Fig. S5), but in some cases also have a limited number of measurements.

Next, we addressed the question of how much of the variation in *g*_m_ can be explained by these two most important anatomical traits. We applied a parsimonious generalised linear model to predict variations in *g*_m_ in response to *T*_cw_ and *S*_c_ accounting for the gamma error distribution present in the *g*_m_ data (please refer to e.g. Fig. 2a). The global model (all data pooled) indicates that these two anatomical traits can explain the variations in *g*_m_ to a reasonable extent across PFTs (*R* ^2^ = 0.48; Table 1), which reflects the consistent global relationship evident in the pairwise plots of *g*_m_ against *S*_c_ and *T*_cw_, respectively (Fig. 3a,b). For individual PFTs, model fits are good for all groups (*R* ^2^ > 0.3) except for annual herbaceous plants (*R* ^2^ = 0.09). In all cases, a greater *g*_m_ was associated with a greater *S*_c_ (β_1_ > 1) and a smaller *T*_cw_ (β_2_ < 1). *S*_c_ could be identified as a statistically significant variable (*P* < 0.05) in all PFTs except for annual herbaceous plants, whereas for *T*_cw_ this was the case for all PFTs except deciduous angiosperms and annual herbs (Table 1).

**Table 1:** Model results of a generalised linear model (glm) of the form log(*g*_m,25_) = β_0_ + β_1_ *S*_c_ + β_2_ *T*_cw_, fitted with a gamma error distribution and a log‐link function.

	No. of studies	No. of measurements	Model coefficients			Model *R* ^2^
			exp(β_0_)	exp(β_1_)	exp(β_2_)	
All plant functional types	50	295	0.128 (0.104, 0.158)***	1.050 (1.038, 1.063)***	0.096 (0.069, 0.133)***	0.48
Ferns	4	46	0.045 (0.025, 0.081)***	1.106 (1.056, 1.161)***	0.244 (0.097, 0.644)***	0.57
Evergreen gymnosperms	7	85	0.056 (0.037, 0.086)***	1.050 (1.033, 1.066)***	0.391 (0.253, 0.614)***	0.46
Evergreen angiosperms	10	57	0.091 (0.039, 0.216)***	1.046 (1.019, 1.075)**	0.082 (0.012, 0.560)**	0.34
Deciduous angiosperms	5	14	0.039 (0.010, 0.165)**	1.093 (1.034, 1.159)*	0.415 (0.005, 47.029)	0.55
C_3_ perennial herbaceous	10	34	0.254 (0.120, 0.548)**	1.030 (1.004, 1.057)*	0.051 (0.005, 0.590)*	0.46
C_3_ annual herbaceous	27	58	0.203 (0.087, 0.464)***	1.031 (0.997, 1.068).	0.282 (0.012, 7.697)	0.09

The model equation is equivalent to *g*_m,25_ = exp(β_0_) exp(β_1_)^Sc^ exp(β_2_)^Tcw^, therefore the exponential function was applied to the model coefficients to allow their interpretation in the original scale of measurement. An increase of *S*_c_ or *T*_cw_ by one unit means that the expected value of *g*_m,25_ is multiplied by exp(β_1_) or exp(β_2_), respectively. Values in brackets give the 95% confidence intervals of the coefficients. The *R* ^2^ represents McFadden's pseudo *R* ^2^ (please refer to the Materials and Methods section). Significance levels are denoted as follows: *P* ≤ 0.1; *, *P* ≤ 0.05; **, *P* ≤ 0.01; ***, *P* ≤ 0.001.

What causes the large variation of *g*_m_ in C_3_ annual herbaceous plants? It is well established that *g*_m_ is not only controlled by leaf anatomy, but also by biochemical factors such as aquaporin content and CA activity (Flexas *et al*., 2018). However, since these factors are typically not measured or not reported in units that allow their intercomparison across studies, our analysis of biochemical leaf traits was limited to leaf nutrient as well as Rubisco contents. Nutrients integrate a wide range of functions within the leaf and therefore do not allow us to infer the immediate role of biochemical mechanisms on *g*_m_. However, they are the only biochemical leaf traits that are frequently measured and reported in common units across studies.

**Fig. 4:**
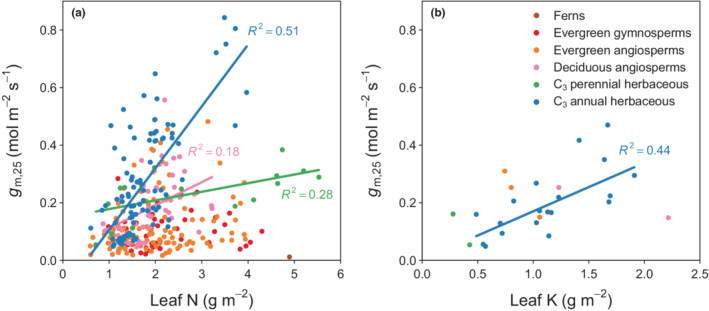
Relationship between mesophyll conductance standardised to 25°C (*g*_m,25_) and (a) leaf nitrogen (N) content, and (b) leaf potassium (K) content. Lines represent robust linear regression fits and were drawn if *P* < 0.05.

Leaf nutrient concentrations for which enough common measurements with *g*_m_ were available comprised leaf nitrogen (N) and potassium (K). Leaf N content per unit leaf area was well correlated with *g*_m,25_ for C_3_ annual herbaceous plants (*R* ^2^ = 0.51, *P* < 0.001) and showed moderate correlations with perennial herbaceous and deciduous angiosperm species, but not for evergreen woody plants (Fig. 4a). C_3_ annual herbs and grasses also show a higher *g*_m,25_ for the same amount of leaf N and a steeper slope, that is, a stronger increase in *g*_m,25_ for a given increase in leaf N compared with other PFTs. Leaf K content per area was also positively related to *g*_m,25_ in leaves of C_3_ annual herbaceous plants across studies (*R* ^2^ = 0.44, *P* < 0.01). The limited availability of K content measurements did not allow us to investigate this relationship in other PFTs. While the number of concomitant *g*_m_ and leaf K content measurements as depicted in Fig. 4(b) is relatively low (*n* = 19), the relationship emerged across unstressed plants from 12 independent studies and is not merely the consequence of an individual experiment.

We next investigated whether the existing relationship between *g*_m,25_ and leaf N was primarily caused by the N allocated to the enzyme Rubisco, which accounts for *c*. 20% of leaf N and constitutes the largest N pool in leaves (Evans & Clarke, 2019). A relationship between *g*_m,25_ and Rubisco content would also indicate a coordination between *g*_m_ and photosynthetic capacity (*V*_cmax_), the two main variables which in combination determine the drawdown from *C*_i_ to *C*_c_. Therefore, we tested whether *g*_m_ and *V*_cmax_ are coordinated in a way to keep *C*_c_, the available CO_2_ concentration for carboxylation, or the ratio *C*_c_ : *C*_a_ relatively constant under unstressed conditions.

**Fig. 5:**
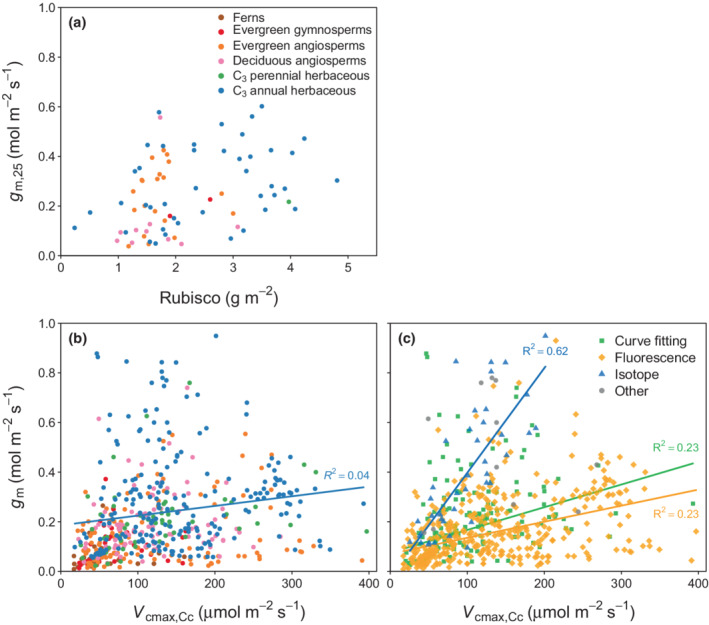
Relationship between mesophyll conductance standardised to 25°C (*g*_m,25_) and (a) leaf Rubisco content, (b) maximum carboxylation rate derived from *A*_n_–*C*_c_ curves (*V*_cmax,Cc_) and (c) same as (b) but data grouped by the measurement method applied. *g*_m_ and *V*_cmax,Cc_ in panels (b, c) were measured at the same temperature. Lines represent robust linear regression fits and were drawn if *P* < 0.05.

In contrast with *g*_m,25_ and leaf N, we have found no statistically significant relationship (*P* > 0.05) between *g*_m,25_ and leaf Rubisco content (Fig. 5a). The data also revealed that *V*_cmax,Cc_, the ‘true’ carboxylation capacity of Rubisco derived from *A*_n_–*C*_c_ curves, is generally unrelated to *g*_m_ across and within PFTs (Fig. 5b) with a large scatter in the reported *V*_cmax,Cc_ for any given *g*_m_. Herbaceous annual plants show a statistically significant positive relationship between *g*_m_ and *V*_cmax,Cc_ but only a weak correlation. The lack of coordination between *g*_m_ and *V*_cmax,Cc_ further results in a wide range of *C*_i_ − *C*_c_ across species and a poorly constrained *C*_c_ : *C*_a_ ratio in unstressed leaves (Fig. 1d,f).

We further investigated whether and how the presented relationship between *g*_m_ and *V*_cmax,Cc_ differs across the three main measurement methods of *g*_m_ (carbon isotopes, chlorophyll fluorescence and curve fitting). We found that *g*_m_ measured with the carbon isotope technique showed a significantly higher correlation with *V*_cmax,Cc_ compared with data measured with the fluorescence or curve fitting methods, in which a strong relationship between *g*_m_ and *V*_cmax,Cc_ is absent (Fig. 5c). The comparison further revealed that the ratio of *g*_m_ to *V*_cmax,Cc_ is greater when measured with the isotope method compared with the fluorescence method.

## Discussion

### Leaf structural and anatomical controls on mesophyll conductance

We conducted a literature analysis of mesophyll conductance (*g*_m_) measurements with the aim of identifying traits that affect *g*_m_ across and within PFTs. We found that leaf structural properties such as LMA, leaf thickness, leaf density and leaf porosity were poorly associated with *g*_m_ for any PFT. This lack of association most probably reflects the integrative nature of these traits. For example, LMA is a product of leaf thickness and density, both of which can vary due to modifications in different underlying traits such as *T*_cw_ or *S*_m_ (Poorter *et al*., 2009; Onoda *et al*., 2017) with potentially opposing effects on *g*_m_ (Onoda *et al*., 2017). By contrast, anatomical traits such as *S*_c_ and *T*_cw_ are expected to have a much more direct effect on *g*_m_ and have previously been identified as important anatomical determinants for leaf internal CO_2_ transfer in all plant groups (Tosens *et al*., 2016; Ouyang *et al*., 2017; Xiong *et al*., 2017; Veromann‐Jürgenson *et al*., 2020). In this analysis, *T*_cw_ and *S*_c_ explained approximately half of the variation in *g*_m_ globally (i.e. all data pooled) as well as within most PFTs, including ferns, evergreen gymnosperms and perennial herbs. Nonetheless, other leaf anatomical traits for which we did not have sufficient data might also play an important role for *g*_m_. Differences in cell wall composition and associated changes in effective cell wall porosity (porosity/tortuosity) have been shown to affect *g*_m_ (Ellsworth *et al*., 2018; Carriquí *et al*., 2020; Flexas *et al*., 2021), and may explain the observed variations in *g*_m_/*S*_c_ for a given *T*_cw_ (Evans, 2021). Notably, our results indicate a much smaller role of leaf anatomical traits in herbaceous annuals compared with other PFTs, which suggests a higher relative importance of traits other than leaf anatomy in this group.

The distinction between leaf structural and leaf anatomical traits also allows some insights into the relative importance of gas and liquid‐phase diffusion components of *g*_m_. Leaf structural traits such as leaf and mesophyll thickness, leaf density, and in particular leaf porosity are expected to be related to CO_2_ transfer conductance in the gaseous phase, whereas anatomical traits such as *T*_cw_ and *S*_c_, but also biochemical factors play a role mainly for CO_2_ transfer in the liquid phase (Niinemets & Reichstein, 2003; Nobel, 2020). The fact that none of the structural leaf traits were related to *g*_m_ within or across PFTs indicates that CO_2_ diffusion in the gas phase is of minor importance compared with CO_2_ transfer in the liquid phase. This view is supported by modelling studies, which assign the largest fraction of the total resistance to the liquid phase throughout PFTs (Tomás *et al*., 2013; Peguero‐Pina *et al*., 2016; Du *et al*., 2019; Carriquí *et al*., 2020).

### Leaf biochemical controls on mesophyll conductance

We found that *g*_m_ is strongly correlated with leaf N and K contents in herbaceous annual plants, moderately correlated with leaf N content in herbaceous perennials and deciduous angiosperms, but uncorrelated with leaf N content in woody evergreens. These findings mirror the current state of the literature. While there is generally a positive association between nutrients and *g*_m_ in woody evergreens, this is in many cases not significant (Warren, 2004; Bown *et al*., 2009; Battie‐Laclau *et al*., 2014). However, experimental studies focusing on nutrient effects in woody evergreens are also less common than those studying herbaceous species. For herbaceous annuals there is strong evidence that leaf macro‐nutrients have positive effects on *g*_m_. This is not only apparent for unstressed leaves across studies (Fig. 4), but it is also a common observation within studies that have considered nutrients as treatment factors. Studies that supplied plants with varying amounts of N usually find a positive association between leaf N content and *g*_m_ in herbaceous plant species (Yamori *et al*., 2011; Li *et al*., 2013; Hu *et al*., 2019; Cai *et al*., 2020). Similarly, higher leaf K content is associated with higher *g*_m_ in a wide range of studies (Lu *et al*., 2016, 2019; Hou *et al*., 2018; Hu *et al*., 2019; Xie *et al*., 2020).

The question remains what underlying biochemical mechanisms cause the clear positive relationship between *g*_m_ and leaf N and K content in herbaceous annuals? As leaf N and K control a vast spectrum of biochemical functions inside the leaf (Wang *et al*., 2016; Evans & Clarke, 2019) we could not attribute leaf N and K content to individual biochemical mechanisms that directly affect *g*_m_. Biochemical factors that have long been suggested as possible determinants for *g*_m_ are aquaporins and CA (Hanba *et al*., 2004; Warren, 2008), proteins that regulate CO_2_ transfer through cell membranes and through the cytosol and the chloroplast stroma, respectively. A positive effect on *g*_m_ has been demonstrated for aquaporins (Hanba *et al*., 2004; Flexas *et al*., 2006; Perez‐Martin *et al*., 2014; Xu *et al*., 2019; but see Kromdijk *et al*., 2020) and to a lesser extent for CA activity (Perez‐Martin *et al*., 2014; Momayyezi & Guy, 2017). There is further limited evidence that leaf N and K are positively associated with the expression of aquaporins (Armengaud *et al*., 2004; Wang *et al*., 2016; Ding *et al*., 2018; Zhu *et al*., 2020) as well as CA activity (Makino *et al*., 1992; Mohammad & Naseem, 2006; Siddiqui *et al*., 2008). However, contrasting results have been reported (Ding *et al*., 2016) and clearly a better understanding of nutrient effects on leaf biochemical functioning is needed (Gao *et al*., 2018). Effects of leaf N on *g*_m_ may also be indirect through changes in leaf anatomical traits as well as photosynthetic capacity with leaf N content. However, the fact that neither leaf anatomy nor Rubisco content could explain a large proportion of the variance in *g*_m_ in herbaceous annual plants points to leaf biochemical factors associated with membranes and cytosol as well as stromal components as more important regulators of *g*_m_ in this group.

### Relative importance of anatomical and biochemical factors across PFTs

Our findings provide strong indications that biochemical and anatomical factors are of contrasting importance for *g*_m_ across PFTs. We found that anatomical factors explain a substantial fraction of the variation in *g*_m_ in all PFTs except annual herbaceous plants. In the latter group, only leaf nutrients could be related to *g*_m_, which in turn were less relevant in other PFTs. Therefore, our results suggest that in annual herbaceous plants leaf biochemical mechanisms associated with leaf nutrients are relatively more important in explaining *g*_m_ across species compared with leaf anatomical traits. Although these results do not imply that biochemical factors constitute a relevant mechanism solely in annual herbs, they suggest that their relative importance for explaining *g*_m_ is higher in this plant group compared with other PFTs. A more prominent role of nonanatomical components for *g*_m_ in annual herbs compared with leaf anatomical features is also suggested by leaf‐level modelling analyses (Tomás *et al*., 2013; Tosens & Laanisto, 2018), and it would be relevant to investigate in how far phylogenetic effects contribute to these differences. Studies looking at changes in *g*_m_ over the course of an experimental treatment (e.g. drought or nutrient stress) have further provided evidence of PFT‐dependent variations in the share of anatomical and biochemical controls on *g*_m_. While in some cases changes in *g*_m_ were linked to leaf anatomical traits (Lu *et al*., 2016; Xie *et al*., 2020), other studies have argued that variations in *g*_m_ could be better explained by changes in leaf biochemistry (Hanba *et al*., 2004; Miyazawa *et al*., 2008; Xiong & Flexas, 2021), which is more in line with the findings in this study.

### Implications for enhancing photosynthesis

We analysed published data on photosynthetic limitation as defined by Farquhar & Sharkey (1982), an approach that quantifies the photosynthetic limitation caused by *g*_m_ (*L*_m_) given the coexisting limitations imposed by stomata and leaf photosynthetic capacity. Our results emphasise the possible underestimated potential of *g*_m_ for improving photosynthesis by increasing the available CO_2_ concentration at the sites of carboxylation (*C*_c_). This does not only apply to PFTs with inherently low CO_2_ diffusion conductance, but also to herbaceous annual crops, which show the highest *g*_m_ values of all plant types. Representative *g*_m_ values measured in crop species under unstressed conditions (*c*. 0.26 mol m^−2^ s^−1^) imply a limitation to photosynthesis that can be attributed to *g*_m_ of *c*. 20% under ambient CO_2_ concentrations, but this percentage is likely to be higher in some crop species such as rice, bean, tomato or soybean, which show a relatively low *g*_m_ compared with other crops (Fig. S3). The values compiled here further suggest that an increase of *g*_m_ in crops from 0.26 to 0.42 mol m^−2^ s^−1^ (i.e. from the median to the 75^th^ percentile) has the potential to increase photosynthesis by *c.* 8%, with probable positive effects on crop productivity and yields (Xu *et al*., 2019).

How could an increase in *g*_m_ be achieved in annual crops? Based on our results we argue that plant engineering and breeding efforts targeting biochemical leaf properties (Groszmann *et al*., 2017; Lundgren & Fleming, 2020) are more promising than those that focus on anatomical traits (e.g. engineering for thinner cell walls) (Tholen *et al*., 2012), which would probably have limited effects on *g*_m_ in crops. The most promising factors in that respect are those that facilitate CO_2_ transfer across membranes, cytosol and stromal components such as CA activity and aquaporins. The fact that good correlations between *g*_m_ and light‐saturated photosynthesis have been observed throughout the literature (e.g. Evans *et al*., 1994; Centritto *et al*., 2009; Fullana‐Pericàs *et al*., 2017) also suggests that increases in Rubisco content and/or Rubisco properties such as *V*_cmax_ are able to increase *g*_m_. However, we found that the true carboxylation capacity of Rubisco, that is the *C*_c_‐based *V*_cmax_ (*V*_cmax,Cc_), does not or only poorly correlate with *g*_m_ across and within PFTs, which corroborates findings from earlier data compilations (Ethier & Livingston, 2004; Warren & Adams, 2006). Consequently, an increase in *V*_cmax,Cc_ (and therefore a higher rate of CO_2_ consumption) is not necessarily concomitant with an enhanced supply rate of CO_2_ through increased *g*_m_, an observation that was supported by a widely varying *C*_i_ − *C*_c_ and *C*_c_/*C*_a_ in unstressed plants. These results imply that plant engineering efforts that focus solely on enhancing Rubisco catalytic rate (Galmés *et al*., 2019), one of the suggested main pathways for improving photosynthesis (Long *et al*., 2006), are likely to be less effective in increasing photosynthetic productivity than parallel increases in both *g*_m_ and Rubisco activity, which would enhance both CO_2_ demand and supply.

### Pathways for future research

The dataset presented here gives unprecedented insights into the extent of photosynthetic limitation imposed by *g*_m_ as well as its anatomical and biochemical controlling factors across PFTs. The results from this meta‐analysis further have the potential to motivate future research activities. Our findings strongly suggest that the sources of disagreement among measurement methods of *g*_m_ deserve further scrutiny. In particular the causes of the differences between the two widely established isotope and fluorescence methods and their relationship with *V*_cmax,Cc_ need to be resolved to not critically confound findings as reported here and in other meta‐analyses (e.g. Onoda *et al*., 2017; Gago *et al*., 2019; Ren *et al*., 2019), which usually pool *g*_m_ measurements across methods. Similarly, the effects of other sources of variation in the data, such as species, growth conditions or growth stages need to be investigated in more detail.

We further argue that a better mechanistic understanding of the factors underpinning the results reported here are urgently required. Particularly the potential links between leaf nutrients and biochemical mechanisms affecting CO_2_ diffusion inside leaves need to be better understood. We suggest that controlled experiments in combination with the latest leaf‐level modelling approaches (Tholen & Zhu, 2011; Xiao & Zhu, 2017) will be best suited to elucidate the role of individual biochemical and anatomical leaf traits for *g*_m_ across PFTs.

## Author contributions

JK designed the study, compiled and analysed the data and wrote the manuscript. JRE contributed to data analysis. L‐LV‐J, TT and ÜN contributed data. All coauthors (MC, ÜN, TT, L‐LV‐J, CW, SZ) contributed to the interpretation of the data, the presentation of the results, and the writing of the manuscript.

## Supporting information


**Fig. S1** Number of studies per year and measurement methods applied to determine *g*_m_.
**Fig. S2** Violin plots of *g*_m,25_ for different PFTs by measurement method.
**Fig. S3** Violin plots of *g*_m,25_ for crop species with at least five individual measurements.
**Fig. S4** Relationships between *g*_m,25_ and leaf structural traits.
**Fig. S5** Relationships between *g*_m,25_ and leaf anatomical traits.
**Table S1** Overview of leaf‐level traits reported in this study.
**Table S2** Statistics of the main results in this study with *g*_m_ standardised by two different temperature response functions.
Please note: Wiley Blackwell are not responsible for the content or functionality of any Supporting Information supplied by the authors. Any queries (other than missing material) should be directed to the *New Phytologist* Central Office.

## Data Availability

All data presented in this manuscript are publicly available via Figshare (Knauer *et al*., 2022).
