# Supplementary material for: Contrasting anatomical and biochemical controls on mesophyll conductance across plant functional types
Source: New Phytol. 2022 Aug 2;236(2):357–68. doi: 10.1111/nph.18363 (PMC9804998; doi:10.1111/nph.18363)
Supplement: Supplementary file 1 — Fig. S1 Number of studies per year and measurement methods applied to determine g m. Fig. S2 Violin plots of g m,25 for different PFTs by measurement method. Fig. S3 Violin plots of g m,25 for crop species with at least five individual measurements. Fig. S4 Relationships between g m,25 and leaf structural traits. Fig. S5 Relationships between g m,25 and leaf anatomical traits. Table S1 Overview of leaf‐level traits reported in this study. Table S2 Statistics of the main results in this study with g m standardised by two different temperature response functions. Please note: Wiley Blackwell are not responsible for the content or functionality of any Supporting Information supplied by the authors. Any queries (other than missing material) should be directed to the New Phytologist Central Office. [file NPH-236-357-s001.pdf]

New Phytologist

***New Phytologist* Supporting Information**

Article title: Contrasting anatomical and biochemical controls on mesophyll conductance across plant functional types

Authors: Jürgen Knauer, Matthias Cuntz, John R. Evans, Ülo Niinemets, Tiina Tosens, Linda-Liisa Veromann-Jürgenson, Christiane Werner, Sönke Zaehle

Article acceptance date: 30 June 2022

The following Supporting Information is available for this article:

Figure S1: Number of studies per year and measurement methods applied to determine g_m.

Figure S2: Violin plots of $g_{m,25}$ for different PFTs by measurement method.

Figure S3: Violin plots of $g_{m,25}$ for crop species with at least five individual measurements.

Figure S4: Relationships between $g_{m,25}$ and leaf structural traits.

Figure S5: Relationships between $g_{m,25}$ and leaf anatomical traits.

Table S1: Overview of leaf-level traits reported in this study.

Table S2: Statistics of the main results in this study with g_m standardised by two different temperature response functions.

Figure S1: Number of studies per year and measurement methods applied to determine g_m. Year refers to the year in which the study was published in issue, not published online. Studies published in issue after 2020 are not shown.

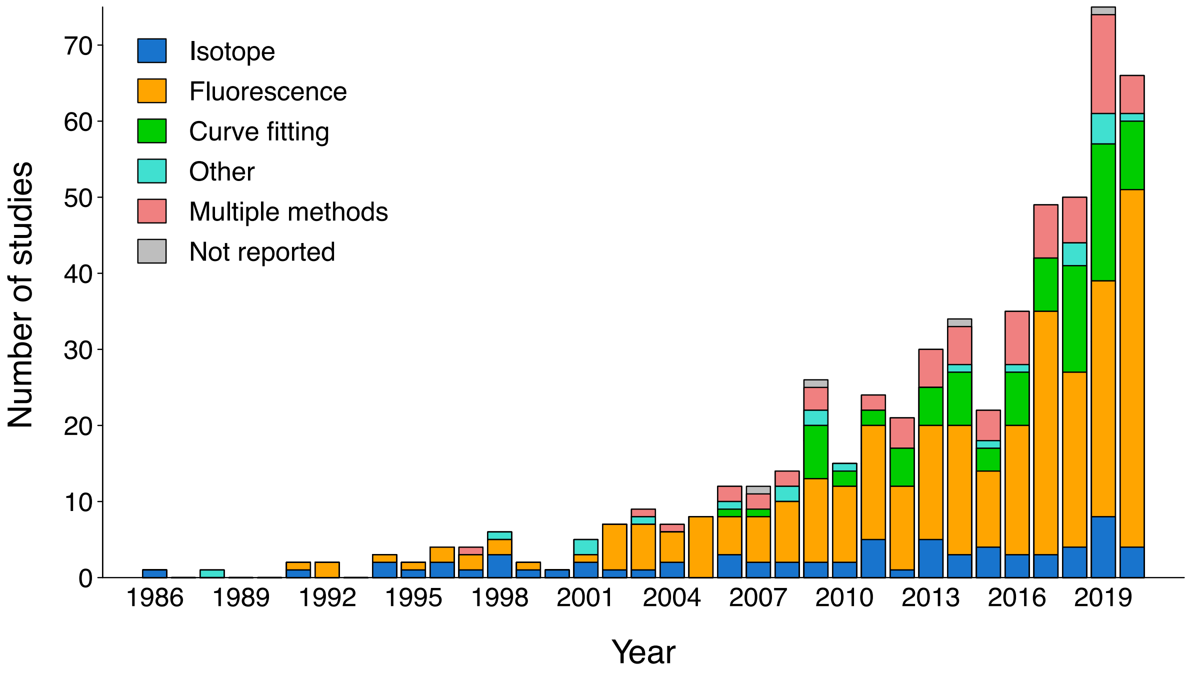

Figure S2: Violin plots showing mesophyll conductance standardised to 25 °C ($g_{m,25}$) for different plant functional types grouped according to measurement method (C: curve fitting, F: fluorescence, I: isotope, other: all other methods pooled). Dots represent group medians, black bars represent the interquartile range, and black lines represent 1.5 times the interquartile range. Shown are only groups with at least seven observations ($n \geq 7$). Letters above the panel indicate differences in the mean according to Dunn's test of multiple comparisons ($p < 0.05$).

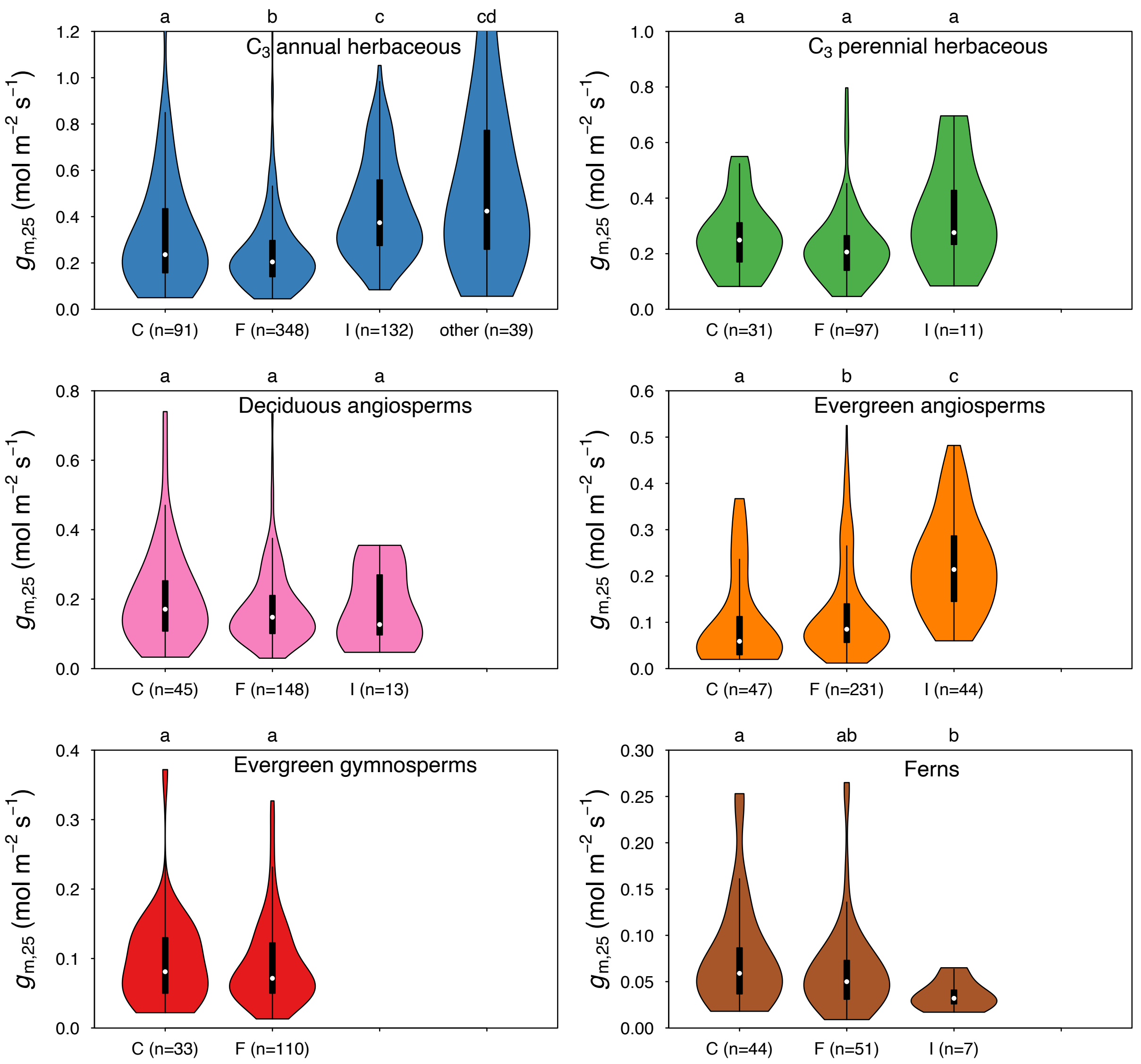

Figure S3: Violin plots showing mesophyll conductance standardised to 25 °C ($g_{m,25}$) for herbaceous crop species with at least five individual measurements. The white dots represent the species median, the black bars represent the interquartile range, the black lines represent 1.5 times the interquartile range, and the dashed grey line represents the median g_m across all measurements in herbaceous crops (0.26 mol m^{-2} s^{-1}). The last two violins show all measurements of annual and perennial crops, respectively, including species with less than five measurements.

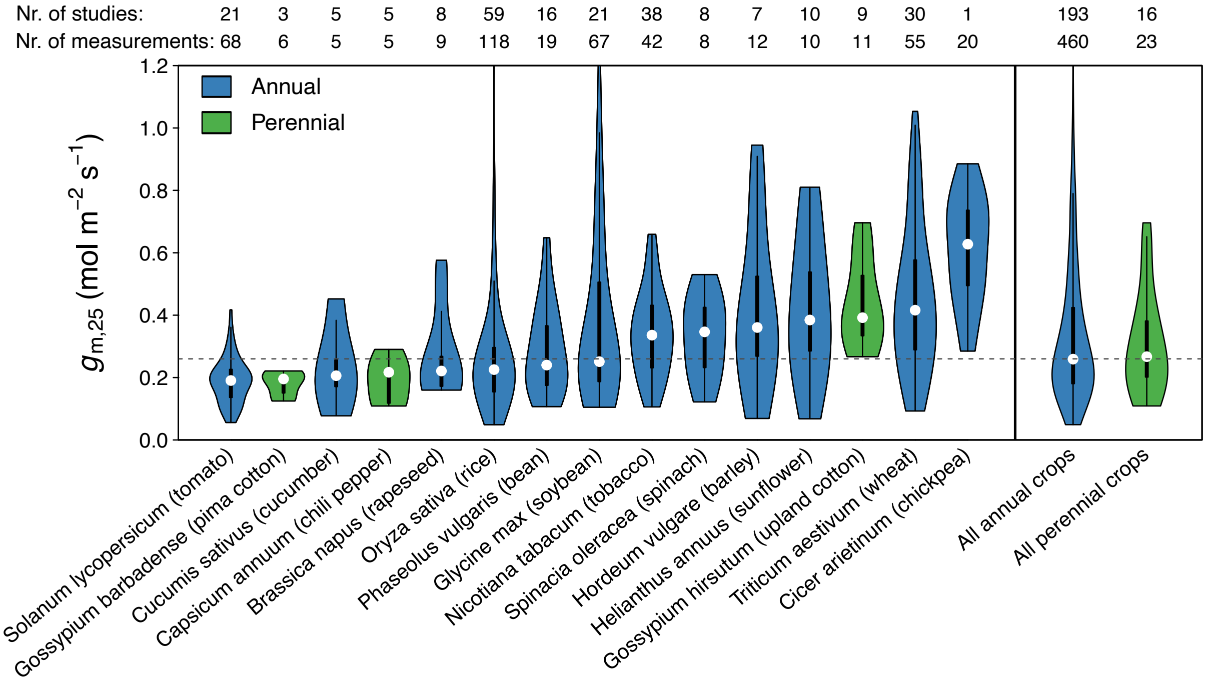

Figure S4: Relationships between mesophyll conductance standardised to 25 °C ($g_{m,25}$) and leaf structural traits: (a) leaf dry mass per area (LMA), (b) leaf density (D_{leaf}), (c) leaf thickness (T_{leaf}), (d) mesophyll thickness ($T_{mesophyll}$), (e) fraction of leaf mesophyll composed of intercellular air spaces (f_{ias}), (f) stomatal length ($L_{stomata}$; abaxial and adaxial combined), (g) stomatal area ($A_{stomata}$; abaxial and adaxial combined), (h) stomatal density abaxial ($SD_{abaxial}$) (i) stomatal density adaxial ($SD_{adaxial}$). Lines represent robust linear regression fits, which are only drawn if $p < 0.05$.

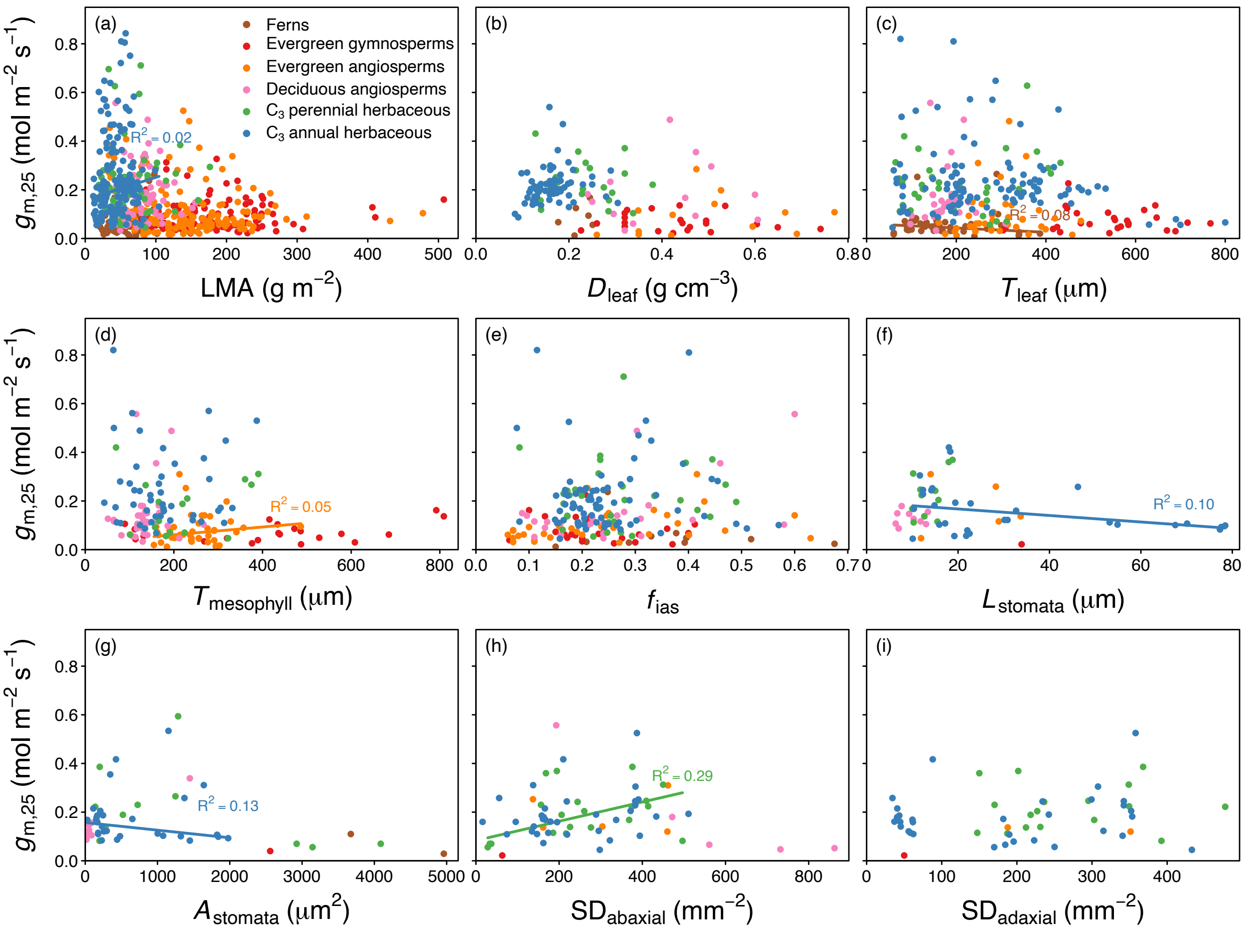

Figure S5: Relationships between mesophyll conductance standardised to 25 °C ($g_{m,25}$) and leaf anatomical traits: (a) surface are of mesophyll cells exposed to the intercellular airspaces per unit leaf area (S_m), (b) ratio of chloroplast surface area exposed to the intercellular airspaces per unit leaf area (S_c) to S_m, (c) cytosol thickness ($T_{cytosol}$), (d) chloroplast thickness ($T_{chloroplast}$), (e) chloroplast length ($L_{chloroplast}$), (f) chloroplast surface area ($S_{chloroplast}$). Lines represent robust linear regression fits, which are only drawn if $p < 0.05$.

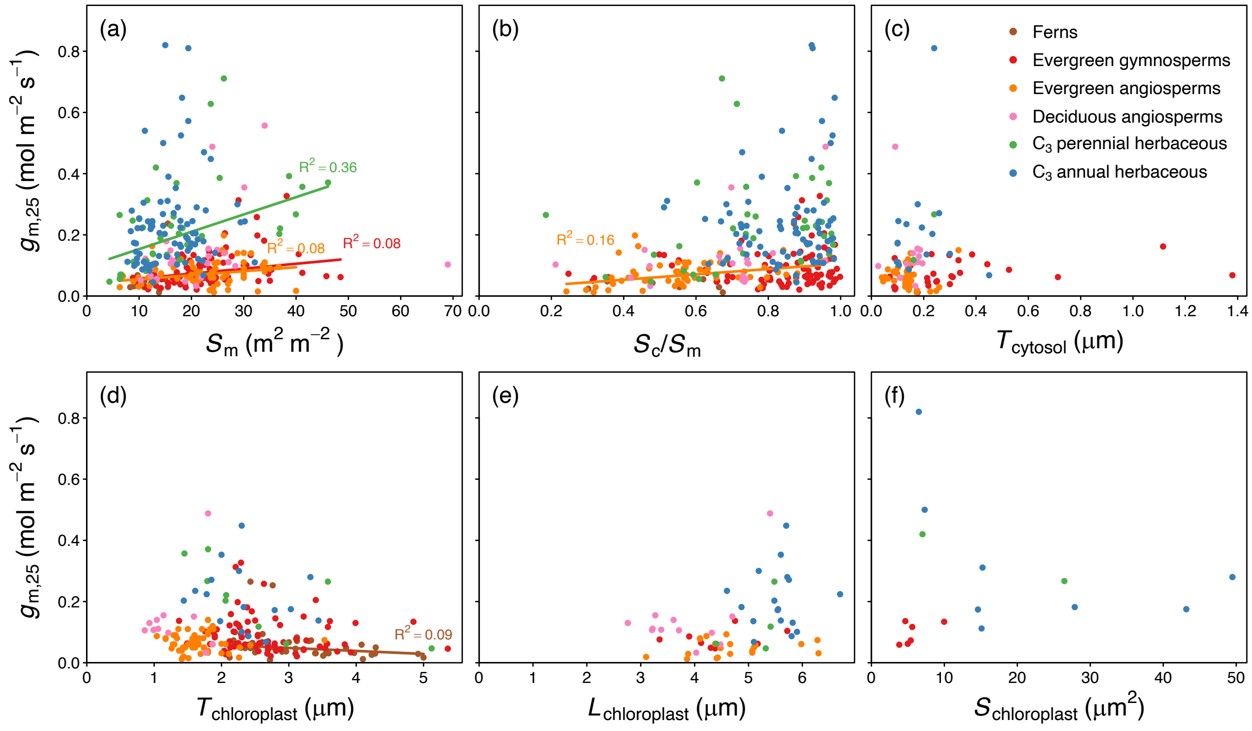

Table S1: Overview of leaf-level traits reported in this study. The number of available data as well as the statistics refer to the dataset after filtering as described in the Methods section.

Trait group	Trait symbol	Definition	Unit	Number of measurements (% of all measurements)	Number of studies (% of all studies)	Min	1st Q	Median	Mean	3rd Q	Max
Leaf physiology	g_m	Mesophyll conductance to CO_2 at measurement temperature standardised to 100 kPa	mol m^{-2} s^{-1}	1337 (100)	476 (100)	0.009	0.089	0.172	0.228	0.305	1.97
	$g_{m,25}$	Mesophyll conductance to CO_2 standardised to 100 kPa and 25 °C	mol m^{-2} s^{-1}	1337 (100)	476 (100)	0.009	0.087	0.164	0.215	0.281	1.50
	A_n	Net photosynthesis rate	µmol m^{-2} s^{-1}	1130 (84.5)	394 (82.8)	1.5	9.0	14.7	16.0	22.4	48.3
	$g_{s,c}$	Stomatal conductance to CO_2	mol m^{-2} s^{-1}	763 (57.1)	250 (52.5)	0.007	0.103	0.174	0.194	0.263	0.955
	$V_{cmax,Cc}$	Maximum carboxylation rate on C_c-basis	µmol m^{-2} s^{-1}	590 (44.1)	220 (47.5)	17.0	63.8	115.4	125.4	164.0	397.0
	C_i	Intercellular CO_2 concentration	µmol mol^{-1}	501 (37.5)	210 (44.1)	131.0	239.7	260.7	261.5	289.6	365.6
	C_c	Chloroplast CO_2 concentration	µmol mol^{-1}	417 (31.2)	173 (36.3)	48.5	128.0	167.9	166.5	200.0	304.0
	L_m	Photosynthetic limitation by g_m	%	46 (3.4)	26 (5.5)	1.1	7.7	18.3	20.4	29.0	55.0
Leaf structure	LMA	Leaf mass per area	g m^{-2}	627 (46.9)	180 (37.8)	11.4	45.1	77.2	100.8	144.5	507.6
	T_{leaf}	Leaf thickness	µm	281 (21.0)	80 (16.8)	58	159	231	268	349	800
	f_{ias}	Fraction of intercellular airspaces	-	198 (14.8)	66 (13.9)	0.06	0.175	0.231	0.253	0.305	0.675
	$T_{mesophyll}$	Mesophyll thickness	µm	155 (11.6)	63 (13.2)	43	132	190	226	279	809
	D_{leaf}	Leaf density	g cm^{-3}	146 (10.9)	26 (5.5)	0.08	0.16	0.21	0.27	0.33	0.77

	$SD_{abaxial}$	Stomatal density (abaxial)	mm^{-2}	65 (4.9)	26 (5.5)	16	162	219	272	383	862
	$L_{stomata}$	Stomatal length	µm	55 (4.1)	19 (4.0)	6.4	11.9	15.7	23.7	28.6	78.5
	$SD_{adaxial}$	Stomatal density (adaxial)	mm^{-2}	52 (3.9)	15 (3.2)	34	82	215	213	322	477
	$A_{stomata}$	Stomatal area	$µm^2$	51 (3.8)	15 (3.2)	13	167	346	929	1348	4961
Leaf anatomy	S_c	Chloroplast surface area exposed to the intercellular airspaces per unit leaf area	$m^2\ m^{-2}$	339 (25.4)	68 (14.3)	1.2	8.7	13.0	13.7	18.0	36.0
	S_m	Mesophyll surface area exposed to the intercellular airspaces per unit leaf area	$m^2\ m^{-2}$	305 (22.8)	66 (13.9)	4.3	13.2	19.3	20.1	24.4	69.0
	T_{cw}	Cell wall thickness	µm	298 (22.3)	52 (10.9)	0.095	0.183	0.307	0.351	0.449	1.220
	$T_{chloroplast}$	Chloroplast thickness	µm	207 (15.5)	26 (5.5)	0.86	1.74	2.26	2.38	2.88	5.36
	$T_{cytosol}$	Cytosol thickness	µm	110 (8.2)	22 (4.6)	0.026	0.129	0.143	0.193	0.215	1.380
	$L_{chloroplast}$	Chloroplast length	µm	61 (4.6)	19 (4.0)	2.76	4.12	4.70	4.76	5.48	6.70
	$S_{chloroplast}$	Chloroplast surface area	$µm^2$	16 (1.2)	7 (1.5)	3.82	5.55	8.64	15.44	18.04	49.48
Leaf biochemistry	Leaf N	Leaf nitrogen content	$g\ m^{-2}$	313 (23.4)	116 (24.4)	0.59	1.38	1.87	2.02	2.31	6.08
	Rubisco	Leaf Rubisco content	$g\ m^{-2}$	77 (5.8)	41 (8.6)	0.24	1.51	1.85	2.38	3.11	10.78
	Leaf K	Leaf potassium content	$g\ m^{-2}$	26 (1.9)	19 (4.0)	0.28	0.73	1.06	1.09	1.37	2.22

Table S2: Model and regression results with two different temperature standardisations of g_m: Bernacchi et al. (2002) as in the main manuscript and Walker et al. (2013). Values in brackets represent 95% confidence intervals (n.s. = non-significant).

	Plant Functional Type	Bernacchi et al. (2002)			Walker et al. (2013)		
Median g_m (Figure 2a)	Ferns	0.050			0.050		
	Evergreen gymnosperms	0.075			0.075		
	Evergreen angiosperms	0.095			0.102		
	Deciduous angiosperms	0.149			0.157		
	C_3 perennial herbaceous	0.210			0.220		
	C_3 annual herbaceous	0.240			0.283		
$g_m – S_c$ relationship (Figure 3a)		Intercept	Slope	Adj. R^2	Intercept	Slope	Adj. R^2
	All PFTs	0.052 [0.029, 0.074]	0.0040 [0.0024, 0.0056]	0.12	0.054 [0.029, 0.080]	0.0046 [0.0027, 0.0065]	0.11
	Ferns	-	-	n.s.	0.024 [0.007, 0.041]	0.0040 [0.0009, 0.0070]	0.18
	Evergreen gymnosperms	0.011 [−0.027, 0.049]	0.0038 [0.0014, 0.0063]	0.23	0.013 [−0.026, 0.052]	0.0038 [0.0013, 0.0062]	0.21
	Evergreen angiosperms	0.007 [−0.010, 0.024]	0.0049 [0.0034, 0.0063]	0.47	0.009 [−0.008, 0.026]	0.0047 [0.0033, 0.0062]	0.46
	Deciduous angiosperms	0.036 [−0.018, 0.089]	0.0049 [0.0017, 0.0082]	0.22	-	-	n.s.
	C_3 perennial herbaceous	0.124 [0.075, 0.172]	0.0067 [0.0039, 0.0095]	0.30	0.128 [0.082, 0.175]	0.0096 [0.0064, 0.0127]	0.43
	C_3 annual herbaceous	-	-	n.s.	-	-	n.s.
$g_m – T_{cw}$ relationship (Figure 3b)		a	b	Adj. R^2	a	b	Adj. R^2
	All PFTs	0.031 [0.022, 0.039]	−0.989 [−1.148, −0.829]	0.33	0.029 [0.021, 0.038]	−1.081 [−1.249, −0.914]	0.32
	Ferns	0.019 [0.011, 0.027]	−0.784 [−1.082, −0.486]	0.14	0.020 [0.012, 0.028]	−0.752 [−1.033, −0.471]	0.12

	Evergreen gymnosperms	0.047 [0.033, 0.062]	−0.752 [−1.063, −0.442]	0.21	0.048 [0.033, 0.063]	−0.762 [−1.085, −0.440]	0.21
	Evergreen angiosperms	0.025 [0.008, 0.041]	−0.952 [−1.566, 0.338]	0.10	0.027 [0.009, 0.046]	−0.858 [−1.468, −0.247]	0.06
	Deciduous angiosperms	-	-	n.s.	-	-	n.s.
	C_3 perennial herbaceous	0.036 [−0.005, 0.078]	−1.02 [−1.634, −0.407]	0.20	0.043 [−0.005, 0.090]	−1.056 [−1.647, −0.465]	0.24
	C_3 annual herbaceous	-	-	n.s.	-	-	n.s.
glm model (Table 1)		R^2			R^2		
	All PFTs	0.48			0.50		
	Ferns	0.57			0.59		
	Evergreen gymnosperms	0.46			0.44		
	Evergreen angiosperms	0.34			0.28		
	Deciduous angiosperms	0.55			0.64		
	C_3 perennial herbaceous	0.46			0.55		
	C_3 annual herbaceous	0.09			0.08		
g_m - leaf N relationship (Figure 4a)		Intercept	Slope	R^2	Intercept	Slope	R^2
	Deciduous angiosperms	0.031 [−0.051, 0.113]	0.080 [0.033, 0.127]	0.18	0.041 [−0.042, 0.125]	0.078 [0.031, 0.125]	0.16
	C_3 perennial herbaceous	0.148 [0.046, 0.251]	0.030 [0.005, 0.055]	0.28	0.085 [0.012, 0.158]	0.051 [0.031, 0.071]	0.63
	C_3 annual herbaceous	−0.109 [−0.224, 0.007]	0.214 [0.148, 0.280]	0.51	−0.077 [−0.183, 0.029]	0.207 [0.146, 0.267]	0.48
g_m - leaf K relationship (Figure 4b)		Intercept	Slope	Adj. R^2	Intercept	Slope	Adj. R^2
	C_3 annual herbaceous	0.002 [−0.097, 0.102]	0.168 [0.048, 0.288]	0.44	0.046 [−0.035, 0.126]	0.123 [0.040, 0.206]	0.35

References

Bernacchi CJ, Portis AR, Nakano H, von Caemmerer S, Long SP. **2002**. Temperature response of mesophyll conductance. Implications for the determination of Rubisco enzyme kinetics and for limitations to photosynthesis in vivo. *Plant Physiology* **130**: 1992–1998.

Walker B, Ariza LS, Kaines S, Badger MR, Cousins AB. **2013**. Temperature response of in vivo Rubisco kinetics and mesophyll conductance in *Arabidopsis thaliana*: comparisons to *Nicotiana tabacum*. *Plant, Cell & Environment* **36**: 2108–2119.
